# Allogeneic tendons in the treatment of malunited lateral malleolar avulsion fractures with chronic lateral ankle instability

**DOI:** 10.1186/s12891-023-06390-1

**Published:** 2023-04-10

**Authors:** Yu Zhang, Xin Wang, Xiaomeng Wang, Jianming Cao, Huijuan Wang, Fengqi Zhang

**Affiliations:** 1 Department of Foot and Ankle Orthopaedics Surgery, The Third Hospital of Hebei Medical University, 139 Ziqiang Road, Shijiazhuang, 050051 Hebei China; 2 Department of Histology and Embryology, Hebei Medical University, Shijiazhuang, 050017 Hebei China

**Keywords:** Chronic lateral ankle instability, Lateral malleolar ligament reconstruction, Allograft tendon, Lateral malleolus avulsion fracture

## Abstract

**Background:**

The aim of this study is to report our institution’s experience regarding the application of allogeneic tendons for the reconstruction of malunited lateral malleolar avulsion fractures with chronic lateral ankle instability.

**Methods:**

This retrospective study included 34 (34 ankles) patients surgically treated for malunited lateral malleolar avulsion fractures with chronic lateral ankle instability from January 2016 to December 2019. All patients underwent allogeneic tendon reconstruction. The pre- and postoperative American Orthopaedic Foot and Ankle Society (AOFAS) scores、Karlsson Ankle Functional Scores (KAFS) and visual analogue scale (VAS) scores were used to evaluate the functional recovery of the ankle joint. The final follow-up, based on radiographic assessment, including talar tilt and anterior talar translation, was performed to evaluate the stability of the postoperative ankle joints.

**Results:**

Thirty-two patients (32 ankles) returned for final clinical and radiologic follow-up at an average of 29 (range 24–35) months and 2 patients (2 ankles) were lost to follow-up. The preoperative talus inclination angle (13.6 ± 1.9°) and anterior displacement (9.6 ± 2.8 mm) were re-examined under X-ray and found to be reduced to 3.4 ± 1.2° and 3.8 ± 1.1 mm, respectively (p＜0.01). The AOFAS scores increased from 58.5 ± 4.0 to 90.9 ± 3.8 and the Karlsson scores improved from 52.2 ± 3.6 to 89.8 ± 4.5, which was obviously better and the difference was statistically significant (P < 0.01). The VAS scores were significantly reduced from a preoperative mean of 6.8 ± 1.0 to 2.8 ± 0.9 postoperatively (p＜0.01).

**Conclusion:**

In this population and with this follow-up, the application of allogeneic tendons to treat malunited lateral malleolar avulsion fractures combined with chronic lateral ankle instability appeared safe and effective.

Acute ankle sprains are a common clinical sports injury, accounting for 15–20% of all sports injuries [1]. The most common type is an ankle varus sprain, which frequently results in a lateral ankle avulsion fracture combined with a peripheral collateral ligament injury [2]. The anterior talofibular ligament (ATFL), calcaneofibular ligament (CFL), and posterior talofibular ligament (PTFL) are the most important lateral collateral ligaments of the ankle. ATFL injuries are present in all patients with chronic lateral ankle pain caused by inversion sprains [3]; CFL injuries account for approximately 50–75%, and PTFL injuries account for less than 10%. Lateral ankle ligament injuries have a high rate of misdiagnosis when diagnosed solely through imaging, however, imaging must be combined with physical examination to reduce the rate of misdiagnosis. Approximately 20–40% of acute ankle sprains result in chronic lateral ankle instability (CLAI), which is frequently accompanied by synovial membrane hyperplasia and articular cartilage damage. The clinical manifestation is ankle joint instability; when walking on a flat road, the ankle joint becomes unstable, frequently swollen, painful, and easily sprained [4]. When a lateral malleolar avulsion fracture occurs and the lateral ankle joint becomes unstable, the restricting structure on the lateral side of the ankle joint relaxes and the load on the medial side of the ankle joint increases, resulting in gradual degeneration of the medial part of the talus and tibial articular surface, causing the ankle joint’s center of rotation to exercise, and traumatic arthritis to occur [5]. As a result, surgical intervention is especially important for patients with chronic lateral ankle injuries. The goal of surgical treatment is to restore ankle joint stability and function while preventing further articular cartilage wear [6].

Direct fixation is challenging and there is a considerable risk of fracture redisplacement when the free bone fragment is tiny [7]. The Brostöm-Gould surgery can be performed to remove the bone fragment and suture the anterior talofibular ligament and extensor retinaculum. Direct fixation was utilized when the free bone fragment was large. But when the free bone fragment is slightly large, It is challenging to directly fix the fractured end during the surgery because it becomes a small bone fragment causing the fixation to become flimsy after the broken end is freshened and the contracted surrounding ligaments cannot be directly sutured by Brostöm-Gould surgery. In order to restore the ankle stability, it is necessary to perform reconstruction surgery at this time [8]. Taking autografts, however, is more labor-intensive for the surgeon and may result in vascular and nerve injuries in the tendon region [9]. The ideal ligament reconstruction should restore ankle stability without affecting the normal biomechanics of the hindfoot, Dierckman [10] believed that using an allogeneic tendon in the treatment of a lateral ankle ligament injury has a good effect in terms of ankle joint stabilization and patient satisfaction.

We hypothesized that connecting the talus to the cuboid during lateral ligament reconstruction would stabilize the midfoot and hindfoot, and ultimately achieve good postoperative results. The aim of this study was to comprehensively evaluate ankle functional recovery after ligament reconstruction by patients’ subjective symptoms and image objective results. We performed allogeneic tendon reconstruction surgeries on 34 patients with malunited lateral malleolar avulsion fractures and CLAI from January 2016 to December 2019. The clinical efficacy and imaging exams were investigated and analyzed and the report is as follows.

## Materials and methods

### Clinical data

This retrospective study included 34 patients (34 ankles) who underwent allograft tendon reconstruction surgery for malunited lateral malleolar avulsion fractures with CLAI, all performed by the same operator. Nineteen (55.9%) of the 34 patients were male, and 15 (44.1%) were female. The mean age at surgery was 43.9 years (range 28–59 years). The left foot was involved in 20 patients (58.8%) and the right foot was involved in 14 patients (41.2%). The average time from the first sprain to the operation was 17 months (range 6–36 months). The mean follow-up was 29 months (range 24–35 months).All patients underwent conservative treatment such as immobilization or painkillers for at least 12 weeks, but the results were not satisfactory. The ankle joints were so unstable that walking on a flat road might cause the ankle joint to be uncomfortable, frequently swollen, and easily sprained. The anterior drawer test and the varus stress test of the ankle joint both returned positive results during the physical examination, which revealed discomfort near the distal end of the lateral malleolus.

This study was approved by the ethics committee of our hospital, and all patients provided informed consent regarding the use of allogeneic tendon and signed the informed consent.

### Inclusion criteria

(1) Those with a history of repeated ankle sprains for 6 months or more have typical symptoms of CLAI, and MRI of the ankle joint indicates that the lateral ligament of the ankle joint is injured; (2) The diameter of the distal bone fragment of the lateral malleolus is about 10–15 mm, which is a closed fracture; (3) Conservative treatment of the initial ankle sprain was ineffective after 12 weeks; (4) The ankle joint was significantly loosened, the anterior drawer test was positive, the lateral ankle joint was tender. The X-ray examination of the varus stress position showed that the talus inclination angle was greater than 9° or the X-ray examination of the anterior drawer stress position showed that the anterior displacement of the talus was more than 5 mm; (5) The ankle joint was not treated by surgery.

### Exclusion criteria

(1) Those whose symptoms were improved after conservative treatment; (2) A history of ankle sprain less than 6 months; (3) The diameter of the lateral malleolar fracture was greater than 15 mm or less than 10 mm, and there was obvious traumatic ankle arthritis; (4) An open ankle fracture; (5) Combined with infection around the ankle joint, medical diseases, etc., that are not suitable for surgery; or (6) Obese, BMI > 28, pregnant or lactating women.

### Treatment methods

**Fig. 1:**
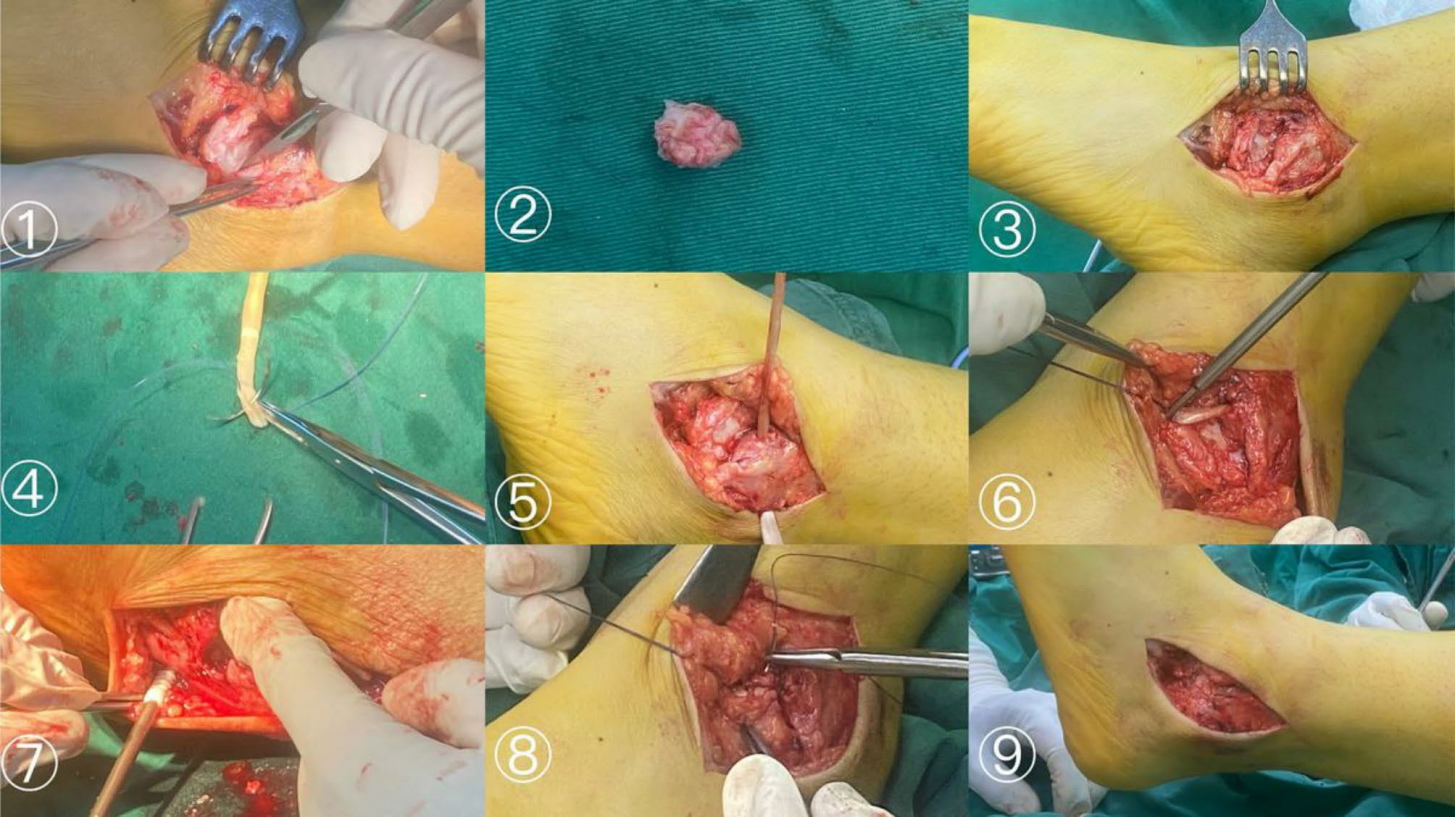
Intraoperative images of the allogeneic tendon transfer procedure: (1) Make an 8 cm incision at the distal end of the lateral ankle joint and separate it layer by layer to the fracture end; (2) Remove free bone fragments; (3) Ligament contractures are seen after removal of bone fragments and cannot be repaired directly; (4) Use an allogeneic tendon and braid; (5) Drill the distal end of the fibula and the tendon is penetrated; (6) Move the tendon to drill the cuboid bone and fix it; (7) Adjust the tension of the tendon and fix it at the drill hole of the calcaneus; (8) Suture the tendon body to the anterior talofibular ligament at the insertion point of the talus and strengthen the suture with the surrounding tissue; 9.The appearance improved after the ligament and tendon are sutured

All operations were performed by the same operator. Combined spinal-epidural anaesthesia was used. After successful anaesthesia induction, the patient was placed in a supine position. A pneumatic tourniquet was placed on the proximal thigh, and routine iodine alcohol disinfection was performed. An arc incision of approximately 8 to 10 cm was made for the muscle course, incision of the skin, subcutaneous and deep fascia, free flaps to both sides, protection of the sural nerve, and exposure of the calcaneofibular ligament and the contracted anterior talofibular ligament. The locally proliferated synovium and scar were removed, the avulsion bone fragment on the anterior talofibular ligament was exposed, and it was carefully freed from the ligament and excised. A hole of 5 mm in diameter was drilled above the lateral malleolar bone fragment from front to back. The soft allograft tendon was soaked in normal saline and iodophor (Beijing Xinkangchen Company) and braided into a graft with a diameter of 4.5 mm and a length of approximately 15 cm, exposing the centre of the cuboid, the talus insertion of the anterior talofibular ligament, and the calcaneofibular ligament. The calcaneal insertion of the ligament was drilled into approximately 15–20 mm with a 5 mm drill. An interface screw (5 mm in diameter, Johnson & Johnson, USA) was used to fix one end of the tendon at the drilled cuboid [11, 12]. The free end of the tendon was inserted into the fibular bone tunnel through a guide wire. In order to avoid postoperative tightness of the lateral ligament resulting in discomfort, the graft tension was adjusted after maintaining the ankle varus by 30°, and the interface screw was used to fix the tendon at the calcaneal burr hole. The ankle joint was placed in the neutral position, the tendon body was sutured to the talus at the ATFL attachment area and simultaneously strengthened with the surrounding tissue, such as anterior border of the lateral malleolus and the inferior pole of the lateral malleolus [11, 12]. The surgical area was flushed with saline, a drainage tube was placed, and the incision was sutured layer by layer. Some surgical steps are shown in Fig. 1.

### Postoperative treatment

The affected limb was externally immobilized in a neutral plaster cast of the ankle joint. After the patient’s vital signs were stable, the patient was assisted in lifting the affected limb to perform straight leg lift and toe function exercises. The drainage tube was removed 24–48 h after the operation and the dressing on the incision was changed every other day. Stitches were removed at 2 weeks and the ankle joint was fixed in a neutral plaster cast. The patient was instructed to be non-weight bearing for 4 weeks after the operation. At 4–8 weeks after the operation, the plaster was removed, the ankle joint brace was fixed and partial weight-bearing was allowed. After 8 weeks, the brace can be removed and the patient can gradually achieve weight-bearing with walking. Patients can be instructed to perform lower limb balance and proprioceptive training.

### Evaluation indicators

The American Orthopaedic Foot and Ankle Society (AOFAS) scores [13] and Karlsson Ankle Functional Scores (KAFS) [14] were used to evaluate ankle function recovery before surgery and at the last follow-up, respectively. The Visual Analogue Scale (VAS) was used to evaluate the subjective proprioception of the patients after surgery [15]. Stress level X-rays were used to measure the talus inclination angle, talus anterior displacement, and surgical complications during the follow-up period.

### Statistical methods

SPSS version 25.0 statistical software(version 24.0 for Windows; SPSS, Inc, Chicago, IL, USA). Was used to process the data, and the AOFAS score, Karlsson ankle function score, VAS score and stress level imaging measurement indexes before the operation and at the last follow-up were analysed. A paired samples t test (x ± s) was used. *P* < 0.05 was considered to be statistically significant.

## Results

**Fig. 2:**
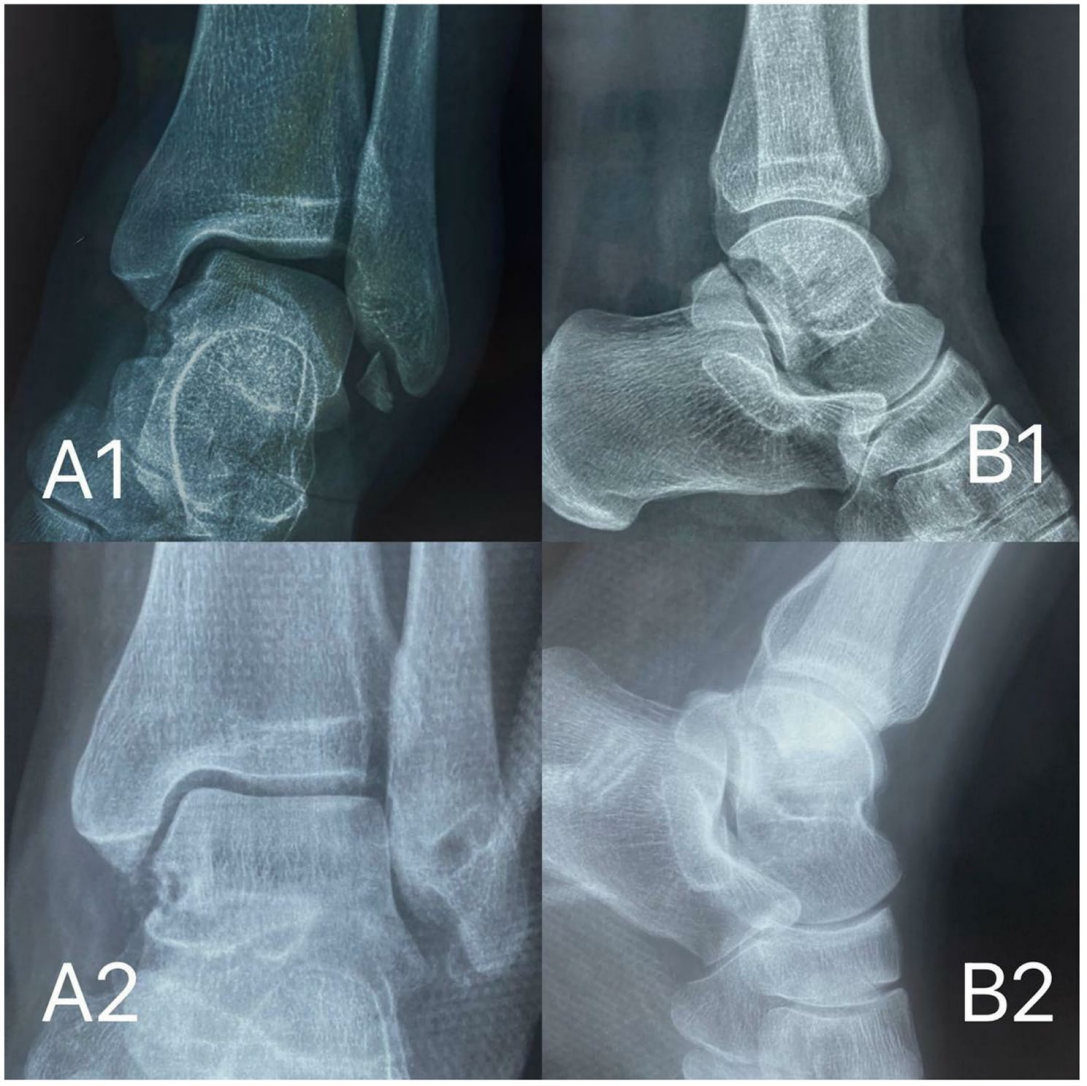
A 35-year-old female patient with right ankle sprain that lasted for 3 years was diagnosed with lateral instability of the right ankle and chronic lateral malleolar avulsion fracture. Preoperative stress level X-rays (A1, B1) and postoperative stress level X-rays (A2, B2)

In this study, 32 patients (32 ankles; 94%) returned for final clinical and radiologic follow-up, while 2 male patients (2 ankles) were lost to follow-up. The incisions of patients(32 ankles) were all healed well after surgery and no vascular-related complications occurred. Thirty-two patients were followed up for 29 (range 24–35) months.After surgery, these patients were able to walk partially weight-bearing at an average of 6 (range 4–8) weeks and fully weight-bearing at an average of 10(range 8–12) weeks.The time to return to strenuous exercise was 6(range 5–7) months. Two patients (6%) had postoperative stiffness of the lateral ankle, restricted varus motion, and no lateral instability of the ankle. Discomfort was alleviated after outpatient-guided functional exercise. Four patients (12.5%) developed mild pain after performing ankle joint weight-bearing postoperatively, which was tolerated. Three patients (9.3%) had a feeling of numbness on the feet and ankles after the operation, which was considered to be caused by pulling the branch of the superficial peroneal nerve and the patients’ abnormal skin sensation gradually disappeared within 2 weeks. At the last follow-up, none of the patients had any symptoms of ankle instability, and the anterior drawer test and varus stress test were also negative. The final AOFAS scores, Karlsson scores and VAS scores were improved compared with those before the operation, and the differences were statistically significant (*p* < 0.01), as shown in Table 1. The preoperative and postoperative imaging examinations of typical cases are shown in Fig. 2.

**Table 1:** Comparison of various indicators in this group of patients before surgery and at the last follow-up (n = 32)

Index	Preoperative	Last follow-up	*t value*	*P value*
AOFAS score (points $\overline x \pm s$)	58.5 ± 4.0	90.9 ± 3.8	-46.225	0.000
Karlsson ankle function score (points $\overline x \pm s$)	52.2 ± 3.6	89.8 ± 4.5	-50.567	0.000
VAS score (points $\overline x \pm s$)	6.8 ± 1.0	2.8 ± 0.9	10.011	0.000
Stress position X-ray talus anterior displacement (mm $\overline x \pm s$)	9.6 ± 2.8	3.8 ± 1.1	24.874	0.000
Stress position X-ray talus inclination angle (°$\overline x \pm s$)	13.6 ± 1.9	3.4 ± 1.2	29.303	0.000

## Discussion

The lateral structures of the ankle joint include the distal end of the fibula and the anterior talofibular, calcaneofibular, and posterior talofibular ligaments [16]. Avulsion fractures of the lateral malleolus belong to the Lauge-Hansen classification of supination and adduct type 1 injuries according to the injury mechanism; that is, when the ankle joint is in the supination position, the talus is subjected to varus stress, which leads to avulsion fractures of the lateral malleolus. The ATFL is the weakest of the lateral collateral ligaments and is damaged in approximately 85% of ankle sprains. It extends from the anterior and inferior border of the fibula to the neck of the talus at an angle of approximately 25° to the horizontal, and its anatomy is often variable [17]. When an avulsion fracture of the lateral ligament insertion occurs, approximately 40% of severe lateral malleolus sprains with torn ligaments lead to CLAI due to improper treatment or lack of awareness of the disease, which in turn leads to traumatic arthritis [18]. Therefore, for displaced avulsion fractures of the lateral malleolus, it is very important to restore the stability of the lateral malleolus as soon as possible.

At present, if there is no obvious displacement of a new lateral malleolar avulsion fracture, conservative treatment can be used to fix the affected limb; for patients with fracture displacement, reduction of the fracture or resection of small bone fragments and ligament repair using the Brostöm-Gould method are the main treatment methods [19]. For malunited lateral malleolus avulsion fractures combined with CLAI, the abnormal stress between the bone fragment and the fibula and the adhesion of the ligament structure often lead to ankle pain and other problems, so surgical treatment is needed. The surgical method depends on the size of the bone fragment. Kim [20] believed that in CLAI patients with free bone fragments, bone fragments with a free bone fragment diameter greater than 10 mm should be reduced, and bone fragments smaller than 10 mm should be removed and the ligament should be repaired. We suggest that large free bone fragments (10–15 mm in diameter) will become small bone fragments after the sclerotic bone is removed from the broken end of the bone fragment, the fixation is not firm, and the surrounding ligament contracture is serious, so fracture reduction and internal fixation cannot be directly performed. Therefore, it is necessary to remove bone fragments and reconstruct the ligament to stabilize the ankle joint to avoid talus cartilage damage and traumatic ankle arthritis [21]. Although autologous tendons such as the gracilis tendon and the tibia-patellar tendon complex [22, 23] can be applied to prevent graft rejection, are simple to acquire and are associated with reduced hospitalization costs [24], anatomical reconstruction surgery of autologous tendons is not effective. The application of autologous tendons causes additional trauma, increases the risk of infection, and may also cause complications in the tendon donor site, therefore sacrificing normal dynamics and affecting stable structures [25]. To avoid the complications of using autologous tendons, Shen [26] advocates the use of allogeneic tendon to reconstruct the lateral ankle joint.

**Fig. 3:**
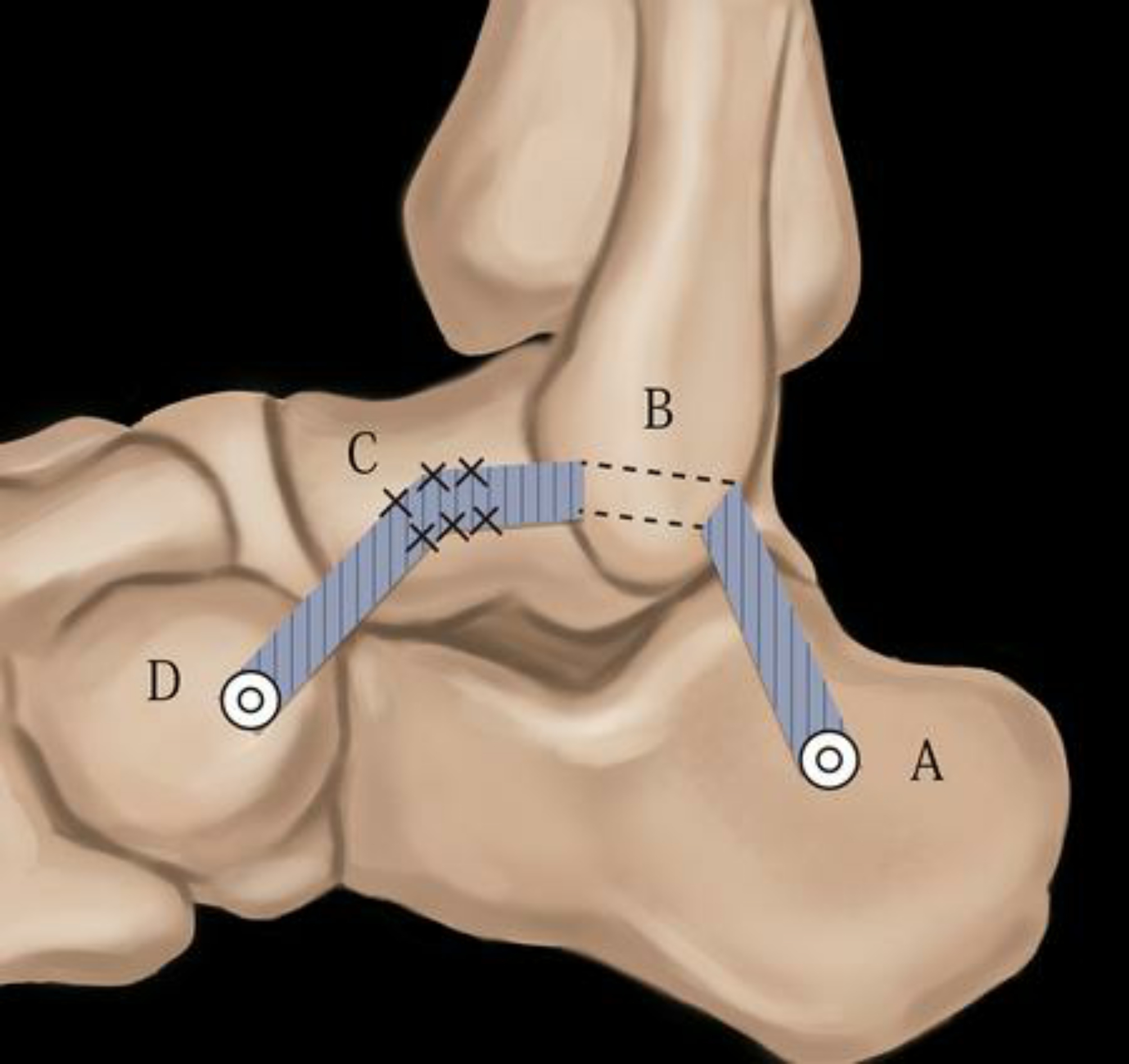
Schematic drawing of the surgery method. A: The anterior half of the allogeneic tendon was fixed into the calcaneal tunnel. B: The allogeneic tendon crossed the fibula. C: The allogeneic tendon was sutured to the attachment area of the ATFL on the talus. D: The allogeneic tendon was fixed into the cuboid tunnel

At present, reconstructive surgery for CLAI restores joint stability by repairing the anterior talofibular ligament and the calcaneofibular ligament [27]. However, Sun [28] believed that approximately 51.4% of patients with CLAI had a bifurcated ligament injury, and a bifurcation ligament injury would lead to lateral laxity of the transverse tarsal joint, resulting in midfoot instability and Chopart joint imbalance [29]. Larsen [30] found that approximately 10–25% of CLAI patients had subtalar joint instability. Hertel [31] found that 15 of them had subtalar joint instability by evaluating adjacent joint function in 20 CLAI patients. Studies have shown an increase in drawer test loosening of the subtalar joint, ranging from 8.13 to 15.43 mm after amputation of the calcaneal ligament [32, 33]. However, we used a Kirschner wire to fix the talus during the operation and found that 11 patients (32%) had increased valgus and anterior displacement distances, which confirmed the existence of subtalar joint instability. Li [34] believed that the stability of the subtalar joint is related to the calcaneofibular ligament, the posterolateral ligament of the talus, and the posterior oblique ligament of the talus. Therefore, surgery in patients with chronic ankle instability simply repairs the lateral ankle ligament, and patients will experience recurrent pain after long-term weight bearing, instability and other symptoms [35]; therefore, we believe that the goals of surgical reconstruction should include restoring the stability of the ankle, subtalar, and Chopart joints. In this study, the fibula and the talus, the fibula and the calcaneus, and the talus and the cuboid were fixed with artificial tendon reconstruction. Connecting the talus to the cuboid is equivalent to strengthening the intertalar calcaneal ligament and the dorsal talar ligament,as is shown in Fig. 3, thereby stabilizing the subtalar joint [12], which better stabilizes the midfoot and hindfoot. All patients in this study had a negative postoperative anterior drawer test, a negative varus stress test and showed no symptoms of ankle instability.

The application of allogeneic tendons to reconstruct the contracture of a lateral malleolar ligament injury has many advantages: (1) Clear exposure and wide range of indications. During the operation, the lateral ligament of the ankle joint can be clearly observed. If it is combined with other tissue damage, it can be repaired at the same time. For injuries with severe ligament contracture, the problem of insufficient tissue can be solved. (2) The trauma is minimal, which reduces the complications of the tendon donor site, and the production technology of an allogeneic tendon is becoming increasingly advanced. After the tendon is processed many times, its antigenicity is reduced, and the biological strength is preserved while avoiding rejection. (3) Surgical connection of the talus to the cuboid can strengthen the ligaments around the subtalar joint and act as a bifurcated ligament, which can better stabilize the midfoot and hindfoot [11].

To prevent excessive tension on the artificial tendon and postoperative iatrogenic ankle stiffness, the length of the graft must be adjusted intraoperatively. Our experience is to maintain the ankle in 30° of varus and tighten the tendon after fixation of the cuboid and then use interface screws to fix the tendon to the calcaneus while maintaining the ankle in a neutral position. However, there were still 2 cases (6.2%) of ankle stiffness and limited varus movement after the operation. The reason for the analysis should be an insufficient ankle function exercise. After an outpatient-guided functional exercise, ankle stiffness was significantly reduced, and the varus movement was improved compared with the previous movement. Four patients (12.5%) developed postoperative ankle pain after weight-bearing, which may be caused by mild traumatic arthritis before surgery. After the operation, 3 patients (9.4%) had abnormal skin paresthesia of the foot and ankle, which gradually diminished within 2 weeks.

This study is a retrospective analysis, and the results may be biased. In this study, the talus and the cuboid were also strengthened and fixed, which could stabilize the subtalar joint and the lateral tarsal transverse joint. The ankle joints of the patients were well stabilized at the postoperative follow-up. However, there are problems such as a small sample size and insufficient scoring criteria. Although the short-term follow-up effect is satisfactory, the long-term effect needs to be observed, and long-term studies need to increase the sample size for further confirmation.

In conclusion, the construction of allogeneic tendons to reconstruct malunited lateral malleolar avulsion fractures with chronic lateral ankle instability is simple, intuitive, worthy of clinical promotion. It is a safe and effective method for patients in our study with malunited lateral malleolar avulsion fractures combined with a ligament injury from the perspective of a short-term clinical follow-up.

## Data Availability

The patients’ data were collected in the Third Hospital of Hebei Medical University. The data that support the findings of this study are available from Fengqi Zhang. but restrictions apply to the availability of these data, which were used under license for the current study, and so are not publicly available. Data are however available from the authors upon reasonable request and with permission of Fengqi Zhang.

## Abbreviations

AOFAS scores: American Orthopaedic Foot and Ankle Society scores
KAFS: Karlsson Ankle Functional Scores
VAS scores: visual analogue scale scores
CLAI: chronic lateral ankle instability
ATFL: anterior talofibular ligament
CFL: calcaneofibular ligament
PTFL: posterior talofibular ligament
